# MRI-based diagnosis and treatment of pediatric brain tumors: is tissue sample always needed?

**DOI:** 10.1007/s00381-021-05148-1

**Published:** 2021-04-05

**Authors:** Jehuda Soleman, Rina Dvir, Liat Ben-Sira, Michal Yalon, Frederick Boop, Shlomi Constantini, Jonathan Roth

**Affiliations:** 1 Departments of Neurosurgery and Pediatric Neurosurgery, Tel-Aviv Medical Center and Dana Children’s Hospital, Tel Aviv University, 6 Weizmann Street, Tel Aviv, Israel; 2 Department of Neurosurgery and Division of Pediatric Neurosurgery, Children’s University Hospital of Basel (UKBB), Basel, Switzerland; 3 Faculty of Medicine, University of Basel, Basel, Switzerland; 4 Division of Pediatric Neurooncology, Tel-Aviv Medical Center and Dana Children’s Hospital, Tel Aviv University, Tel Aviv, Israel; 5 Pediatric Radiology Unit, Dana Children’s Hospital, Tel-Aviv Medical Center, Tel-Aviv University, Tel-Aviv, Israel; 6 Department of Pediatric Hemato-Oncology, Edmond and Lilly Safra Children’s Hospital and Cancer Research Center, Sheba Medical Center, Tel Hashomer, Israel; 7 Department of Neurosurgery, LeBonheur Children’s Hospital, Memphis, TN USA

**Keywords:** Pediatric brain tumors, Magnetic resonance imaging, Surgical biopsy, Image-based diagnosis, Pediatric neurosurgery, Pediatric neuroradiology

## Abstract

Traditional management of newly diagnosed pediatric brain tumors (PBTs) consists of cranial imaging, typically magnetic resonance imaging (MRI), and is frequently followed by tissue diagnosis, through either surgical biopsy or tumor resection. Therapy regimes are typically dependent on histological diagnosis. To date, many treatment regimens are based on molecular biology. The scope of this article is to discuss the role of diagnosis and further treatment of PBTs based *solely* on MRI features, in light of the latest treatment protocols. Typical MRI findings and indications for surgical biopsy of these lesions are described.

## Introduction

Current management of newly diagnosed pediatric brain tumors (PBTs) consists of central nervous system (CNS) imaging, typically magnetic resonance imaging (MRI), followed by tissue diagnosis, through either surgical biopsy or tumor resection. Many treatments to date are based on the molecular profile. For some tumors, however, the surgical risks of a biopsy may not be negligible, and the added value of tissue examination may be marginal. In such cases, the diagnosis may be based only on the MRI findings. The aim of this review is to explore the role of MRI-based diagnosis and treatment of these PBTs in the current oncological era.

## Diffuse intrinsic pontine glioma

**Fig. 1:**
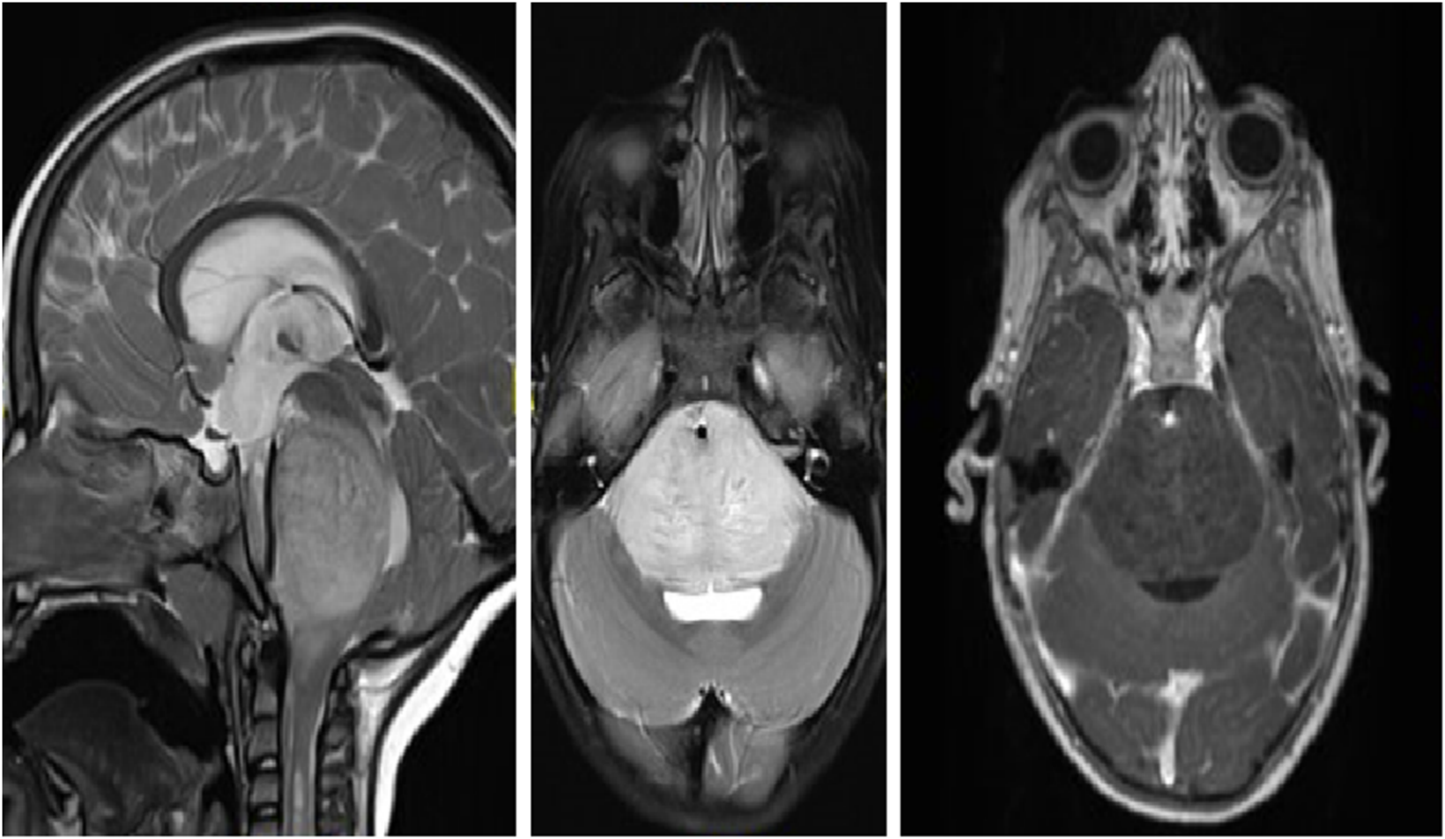
Diffuse intrinsic pontine glioma (DIPG): sagittal (left) and axial (middle) T2-weighted, and axial (right) T1-contrast-enhanced MRI of a 3.5-year-old girl presenting with new-onset cranial neuropathy, motor decline, and headaches. MRI shows a diffuse pontine tumor, with engulfment of the basilar artery, and very mild linear contrast enhancement. A shunt was placed to treat the hydrocephalus. Based on the MRI features, a DIPG was suspected and treated accordingly

Diffuse intrinsic pontine gliomas (DIPGs) are diffuse midline gliomas within the pons and represent 15% of all PBTs and up to 80% of all brainstem tumors in children [1]. No treatment strategy, except for irradiation, has succeeded in significantly improving overall survival over the last decade, while median survival rate is usually <1 year [1–3]. Typical DIPG features on MRI are expansive, infiltrative tumor centered in the pons and originating in the ventral pons, encompassing more than 50% of the axial cross section, and causing diffuse enlargement of the pons (Fig. 1). The tumor often infiltrates the middle cerebellar peduncles (MCP), cranially to the midbrain, caudally to the medulla, and ventrally enveloping the basilar artery. The pattern of extrapontine lesion extension (EPLE) might affect overall survival (OS); infiltration towards the MCP (horizontal MPLE) showed correlation with shorter OS, while vertical MPLE was correlated with longer OS [4]. The tumor is typically hyperintense on T2-weighted imaging and on fluid-attenuated inversion images (FLAIR), while on T1-weighted imaging, the tumor is rather hypo- or isointense. Contrast-enhanced images show either no enhancement of the tumor or mild, linear, and heterogeneous enhancement [2, 5]. Other MRI or imaging modalities, such as MRI spectroscopy (MRS), MRI perfusion, diffusion tensor imaging (DTI), or positron emission tomography (PET), are usually not required for diagnosis of DIPG [5]. However, they might provide from some cases useful information. The choline (Cho)/*n*-acetylaspartate (NAA) ratio on MRS has been shown to have prognostic value for overall survival, similar to increased perfusion at baseline (or at any time) [5]. DTI can help in differentiating DIPG, which displaces the white matter tracts in the brainstem, from demyelinating diseases, where the white matter tracts are truncated and diminished [5]. Increased metabolic activity on PET imaging (typically FDG-PET) shows a correlation with a worse outcome. PET-guided biopsies also seem to increase the diagnostic yield [5].

The standard treatment for DIPG, to date, remains focal radiation therapy to the pons; no surgical or chemotherapeutic strategy has shown improvement in overall survival [2, 6]. Since the mid-eighties, when Epstein et al. published their series condemning biopsy and/or surgical resection of DIPG, imaging features supportive of DIPG, in accordance with typical clinical symptoms, are considered sufficient for diagnosing DIPG and initiating empirical treatment [1, 2, 5–11]. In addition, according to the current World Health Organization (WHO) classification, *K27M* mutant is defined as WHO grade IV, regardless of histological features. Therefore, biopsies are generally reserved for cases with atypical radiological features, such as strongly homogenously contrast enhancing, focal and well-circumscribed, eccentric, or exophytic lesions, which might indicate other pathologies.

Nevertheless, since a large single-institute series of DIPG biopsies was published, showing relatively low rates of transient clinical worsening of symptoms and high diagnostic yield, the trend to perform biopsies in DIPG patients has been growing [2, 3]. These results were partially confirmed by a recently published meta-analysis of 735 brainstem biopsies showing 96% diagnosis success, 6.7% overall morbidity, and 0.6% permanent morbidity and mortality. On the other hand, some authors have recently reported metastatic seeding along the biopsy tract as a rather novel complication of stereotactic biopsies [1, 12]. Over the last 5 years, more and more centers perform biopsies in DIPG patients, mostly within the scope of clinical trials, in order to achieve a better understanding of the molecular characterization of these tumors [3, 10, 13, 14]. Stereotactic biopsy is usually the chosen operative technique [3, 13, 14]. The chosen trajectory (transfrontal vs transcerebellar) varies within the literature and is mostly dependent on the chosen biopsy target within the lesion which influences the needle trajectory [3, 14]. Ideally, biopsies should be taken from both the non-enhancing and enhancing region of the tumor, if possible through one trajectory [3]. It seems that for the surgery itself, no specific expertise is required; however, ideally, these patients should be managed and operated on by specialized pediatric neurosurgeons [13, 14]. The molecular biology of DIPG may have prognostic value for overall survival and tumor response to radiation (e.g., H3.3 K27M mutant may portend a poor response to radiotherapy and average survival of 9 months, while H3.1 K27M mutant may portend a good response to radiotherapy and 15 months average survival) [2]. In addition, targeted therapies based on molecular findings (e.g., TRK inhibitors for NTRK fusion-positive DIPG) will be the focus of future trials searching for a cure for this devastating pathology [13, 15]. For now, biopsies remain investigational and should therefore be offered only within the scope of research protocols [13]. The diagnosis of DIPG based on radiological and clinical features is considered by most to be sufficient, and the current standard of care. However, if after thoroughly discussing the limited benefits a biopsy has to offer, and potential complications, the parents still insist on obtaining a biopsy, it can be done.

### Tectal plate glioma

**Fig. 2:**
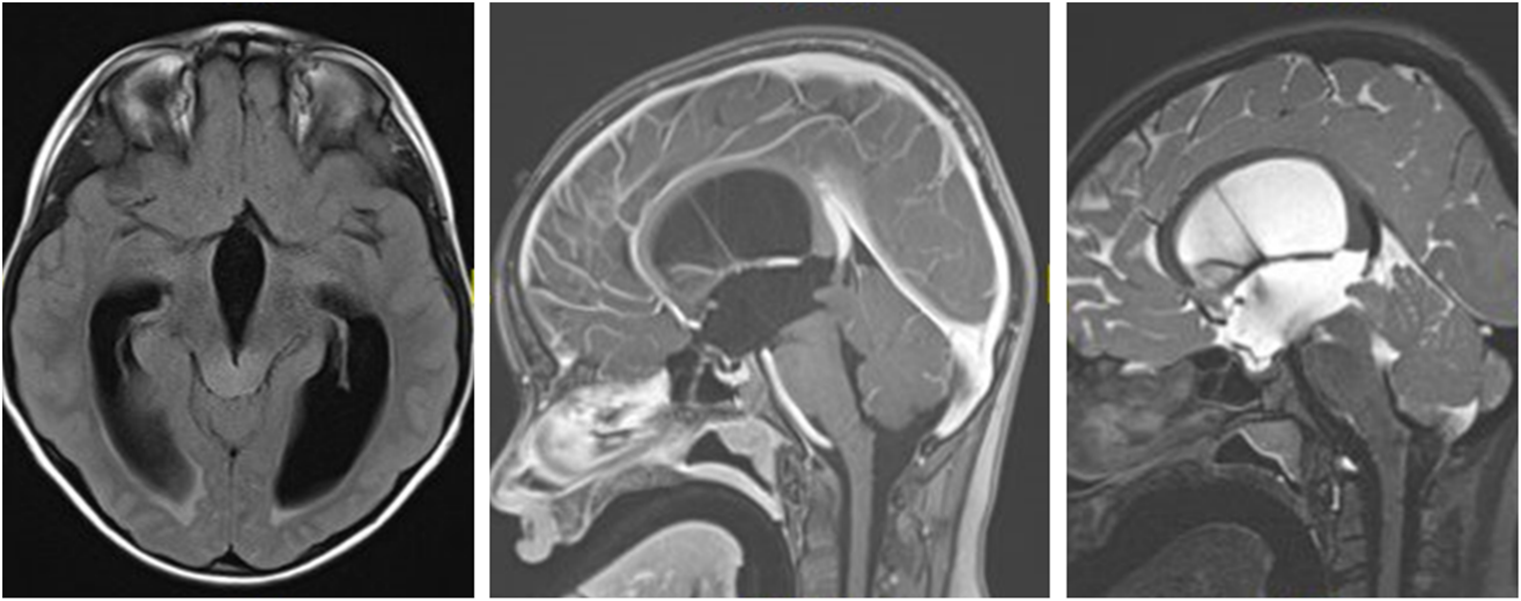
Tectal plate glioma (TPG): axial FLAIR, and sagittal T2-weighted and T1-contrast-enhanced MRI of a 12-year-old girl presenting with obstructive hydrocephalus. MRI shows an isointense lesion on T1-weighted imaging (middle image), without contrast uptake, slightly hyperintense on T2-weighted imaging (right image), leading to a “ballooned” tectum and compression of the aqueduct. The child underwent an ETV and is symptom-free for last 6 years, with no tumor progression

Tectal plate gliomas (TPGs) represent a subset (5%) of midbrain gliomas that are mostly, in contrast to other midbrain gliomas, slow-growing or stable low-grade tumors [16–19]. Histopathology of TPGs is, in the majority of cases, hamartomas or pilocytic astrocytomas, and only very rarely more aggressive tumors such as anaplastic gliomas [19]. Patients present mostly with symptoms of increased intracranial pressure due to obstructive hydrocephalus, secondary to aqueduct compression [16–18, 20]. On MRI, TPGs are typically confined to the tectal plate, manifesting as a “ballooned” tectum (Fig. 2); some lesions extend to adjacent structures such as the tegmentum and thalami [21]. They present in general as iso-/hypointense lesions on T1-weighted images, and hyperintense on T2-weighted images [19, 20]. Contrast enhancement and a cystic component are described in up to 40% and 14% of cases, respectively [19–21]. Patients with TPG have an excellent 10-year overall survival rate of 96%. Tumor volume in around 70% of the cases is ≤ 3cm^3^ at presentation; a volume > 3cm^3^ in combination with contrast enhancement seems to be associated with lower 10-year event-free-survival rates [20].

Existing evidence shows that TPGs very frequently follow a benign clinical and radiographic course, leading in most cases to recommend observation of the lesion. Treatment, beyond that of cerebrospinal fluid (CSF) diversion (endoscopic third ventriculostomy (ETV) or ventriculoperitoneal shunting (VPS)), is rarely required [16, 17, 19]. Based on the largest prospective study to date, more than half of the patients presenting with TPG and treated conservatively will not need any tumor treatment [20]. Indications for treatment, including radiotherapy, chemotherapy, or surgical resection, are not universally agreed upon, but seem to be indicated only when tumor progression or significant progressive neurological symptoms occur. It remains unclear which MRI criteria of TPG might indicate a more aggressive tumor requiring tumor biopsy and/or upfront treatment. However, it seems that tumors > 3cm^3^, with contrast enhancement and cystic changes, are at greater risk for progression, which might, at some point, lead to treatment with or without tumor biopsy [20, 21].

In general, most TPGs are currently managed conservatively, without taking biopsy specimens [16, 17, 20]. While in our practice treatment is usually initiated without obtaining a biopsy, some would insist on collecting a tissue sample before treatment. Lesions protruding into the aqueduct or progressing in size, leading to hydrocephalus, should be considered for a biopsy concurrently with an ETV, especially if they display contrast enhancement or are suspected of being an aqueduct tumor [22, 23]. In cases where tumor progression occurs despite first-line chemotherapy, a biopsy should be obtained. Within the scope of some current (e.g., COG protocol ACNS1833) and planned (e.g., SIOPE LOGICC) study protocols, tissue sampling is mandatory and should be obtained as well.

### Bilateral thalamic glioma

**Fig. 3:**
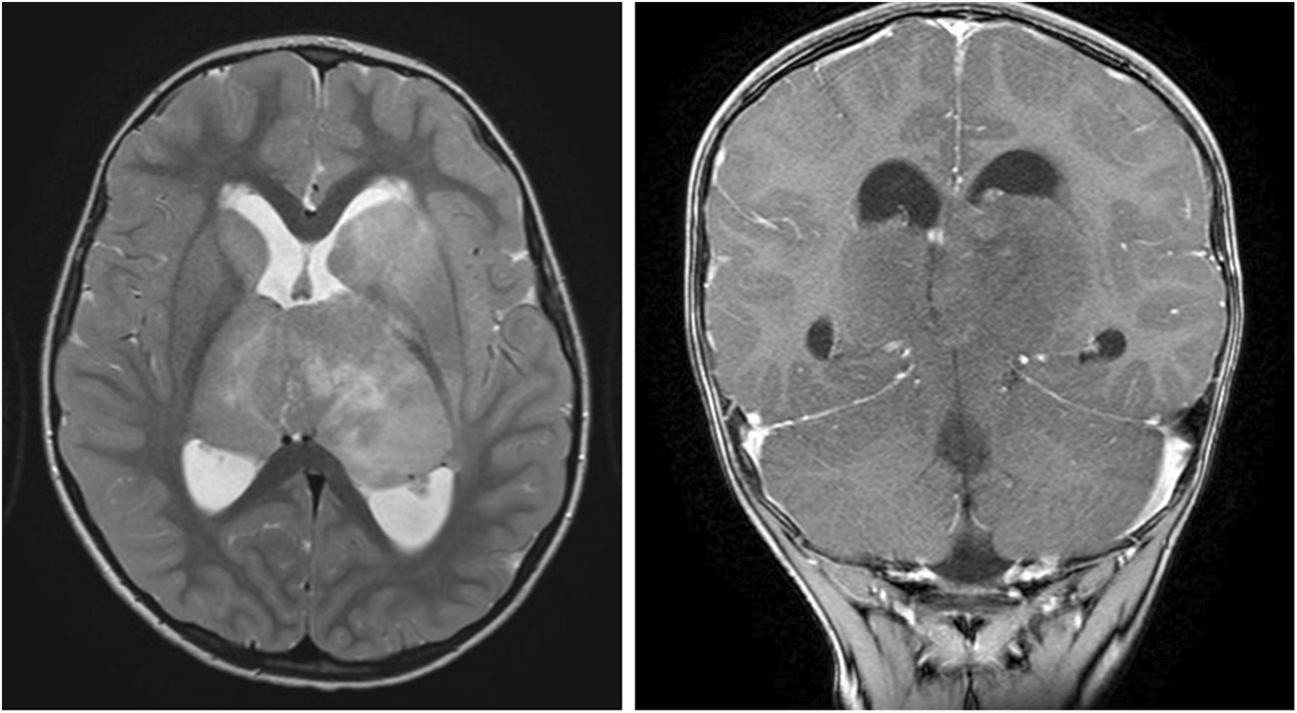
Bilateral thalamic glioma (BTG): axial T2-weighted and coronal T1-contrast-enhanced MRI of a 4-year-old boy presenting with symptoms of increased intracranial pressure. MRI shows a diffuse bithalamic tumor, extending to the basal ganglia (especially on the left). An endoscopic third ventriculostomy and septostomy were performed. Patient succumbed to disease about 1 year later, following oncological treatment

Thalamic gliomas are very rare lesions, occurring in 0.84–5.2% of children with PBTs. Around 15% of thalamic gliomas are bilateral (BTGs) [24–26]. In children, these tumors usually present between the ages of 6 and 9 years, with signs of increased intracranial pressure (compression of the aqueduct or third ventricle), motor or sensory deficits, movement disorders (e.g., tremor, spasticity), mental changes, or cognitive decline [24–26]. The often-mild symptoms, coupled with difficulties in interpreting the neuroradiological findings, which can be similar to encephalitis and neurometabolic disorders, may sometimes be misleading [25, 27]. Typical radiological characteristics of BTG on MRI include bilateral lesions of the thalami with a homogenous aspect, a compact epicenter, the absence of contrast enhancement, hypointense on T1-weighted images, hyperintense on T2-weighted images and FLAIR, and mild or absent perifocal brain edema, often showing growth of the lesions on follow-up MRI (Fig. 3) [24–27]. Overall, imaging features observed in BTG are similar to those in DIPG and all other DMGs. Although rare, some bithalamic lesions show imaging features consistent with low-grade gliomas (e.g., pilocytic astrocytoma), hence, presenting as well-delineated lesions with slow progression and better survival. However, more often, a different pattern, such as infiltrative tumors affecting the thalamus, with a more “DMG appearance” is seen. These lesions tend to evolve with a rapid increase in size and show poor outcome. On MRS, typical findings include decreased NAA concentration and increased Cho concentration, with only a mild peak of lactate concentration. These findings contrast clearly with mitochondrial encephalopathies (e.g., Leigh syndrome), which show significant increases in lactate concentration, and help distinguish BTG from other neurological entities [25, 27]. In addition, BTGs have been described as tumors remaining within the thalamus, respecting the gray/white matter border. However, at times, usually in the later phases of their evolution would they infiltrate other adjacent structures, such as the temporal lobe, brainstem, basal ganglia, and amygdala [28, 29].

Histopathology of these tumors is mostly either low-grade astrocytoma (WHO grade II) or WHO grade III and IV [24, 26]. Surgical resection is not feasible, unlike unilateral thalamic lesions. Based on the scarce literature available, most treating physicians request stereotactic, open, or endoscopic biopsy of BTG before initiating treatment, even though survival rates are very poor and treatment is usually radiotherapy, independent of the histological grading [24–28]. Very recently, a small series of patients with BTG showed that 85% harbor a mutation of the EGFR oncogene [30, 31]. Treatment using targeted kinase inhibitors was initiated in four children with BTG showing promising results [31]. Further data is needed to confirm these results concerning the treatment effect of targeted kinase inhibitors for BTG. Similar to DIPGs, diagnosis of BTGs based on the typical MRI and MRS characteristics is sufficient. Biopsy preceding treatment is, in our opinion, best offered within the scope of research protocols, with the goal to gain better information on tumor biology (e.g., EGFR alterations) and the effect of targeted therapy.

### Optic pathway gliomas in neurofibromatosis type I patients (OPG in NF1)

**Fig. 4:**
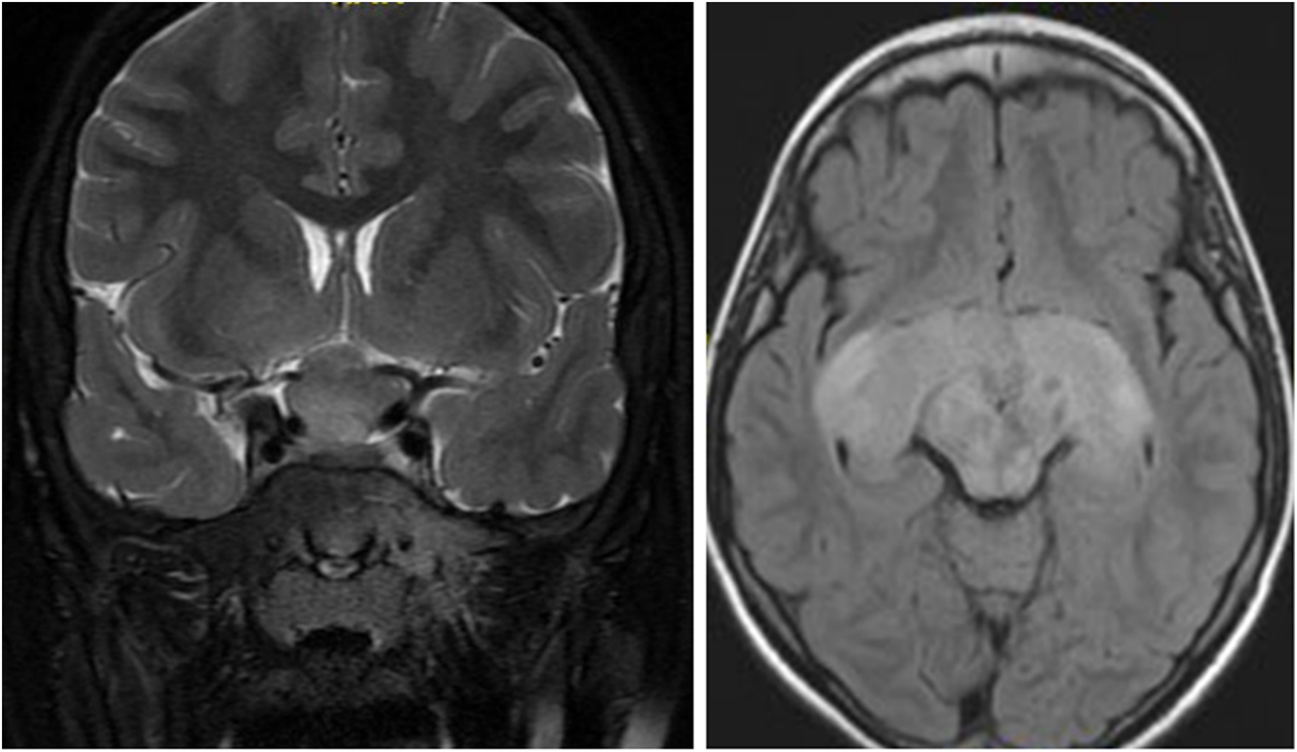
Optic pathway glioma (OPG) in neurofibromatosis type I (NF1): coronal T2-weighted MRI and axial FLAIR MRI of a 9-year-old boy with NF1. MRI shows a typical OPG, involving the optic nerves, chiasm, and optic tracts. Typical NF changes are seen in the mid-brain. The child underwent treatment with vincristine and carboplatin, followed by vinblastine. Over the years, the tumor reduced in size; however, vision continued to deteriorate

OPG is the most common NF1-associated central nervous system tumor, affecting 15–20% of the children with NF1 [32–34]. OPGs occur anywhere along the optic pathway (Fig. 4) [33]. The most common histology is known to be pilocytic astrocytoma [35]. Although the course of OPG in NF1 is usually more indolent compared to other instances, sometimes severe impairment of visual function, hypothalamic abnormalities (including precocious puberty), or potentially life-threatening behavior occurs [32, 36]. Increased risk for tumor progression was seen in patients under the age of 2 years or above the age of 8–10 years, children of female sex, and post-chiasmatic tumors [33]. Baseline MRI was originally not indicated for NF1-OPG screening, since the detection of an asymptomatic tumor rarely changes the management course [33]. However, visual screening is now recommended, including thorough yearly age-appropriate eye exams, in all NF1 children under the age of 10 years, and at least every 2 years thereafter until the age of 18 years [37]. For children in whom reliable visual assessment cannot be performed, such as infants and young children, screening with neuroimaging may play a bigger role [33].

Pathognomonic MRI findings of OPG, in combination with the diagnosis of NF1 and noticeable changes on eye exam, lead to the diagnosis of OPG [35, 38]. Therefore, surgical biopsy has practically no role in tumor diagnosis of OPG in NF1 children [38, 39]. However, biopsy is increasingly indicated once aggressive or transformed tumor behavior is suspected, focused not only on histologic confirmation of the tumor type and its histologic grade but also on genetic alterations, such as *ATRX*, *CDKN2A*, and *TP53*, suggestive of transformed or higher-grade tumors [40]. OPGs characteristically cause an enlargement of the optic nerve(s), chiasm, optic tract, and/or optic radiations seen on MRI [38]. When confined to the optic nerve, they usually have a tubular-fusiform appearance and lead to a downward kink in the mid-orbit [38]. In the chiasm, they often appear as enlargements of the chiasm or as a suprasellar mass, sometimes accompanied by a cystic mass [38]. OPGs are isointense on T1-weighted imaging and iso- to hyperintense on T2-weighted imaging. Contrast enhancement can be heterogeneous; therefore, T2-weighted sequences can often define the tumor borders more accurately [34].

Due to its usually indolent course, upfront therapy in non-NF1 patients is usually not indicated. Nevertheless, the management regime remains unclear; the decision hinges upon factors like patient age, gender, presenting symptoms, and tumor location [33, 41]. Generally, treatment is commenced in patients with clinical deterioration and/or radiological progress, although there can still be controversy over that decision. Chemotherapy with vincristine and carboplatin is chosen by most as the first-line treatment [34]. However, if tumor progression or clinical deterioration occurs even under traditional chemotherapy, molecular targeted treatment (e.g., MEK inhibitors) is commenced at an early stage [40]. Since chemotherapy has been shown to be ineffective for cystic tumor components and has only a marginal positive effect on visual outcome, a tendency exists to declare failure at a rather early stage [33, 42, 43]. In these cases, targeted therapy is initiated at an early stage. In case of mass effect, they may be considered candidates for surgery to drain the cystic portion of the tumor. A phase III clinical trial assessing upfront targeted treatment using selumetinib versus chemotherapy (carboplatin/vincristine) is still in progress (NCT03871257) [44]. For NF1 patients, a biopsy is generally not indicated, even for the detection of BRAF mutation status, before initiating treatment with a MEK 1/2 inhibitor (e.g., selumetinib) for recurrent or progressive tumor growth, and is reserved for cases with suspected tumor transformation or an unusual location and/or presentation [34, 45].

### Subependymal giant cell astrocytoma in tuberous sclerosis

**Fig. 5:**
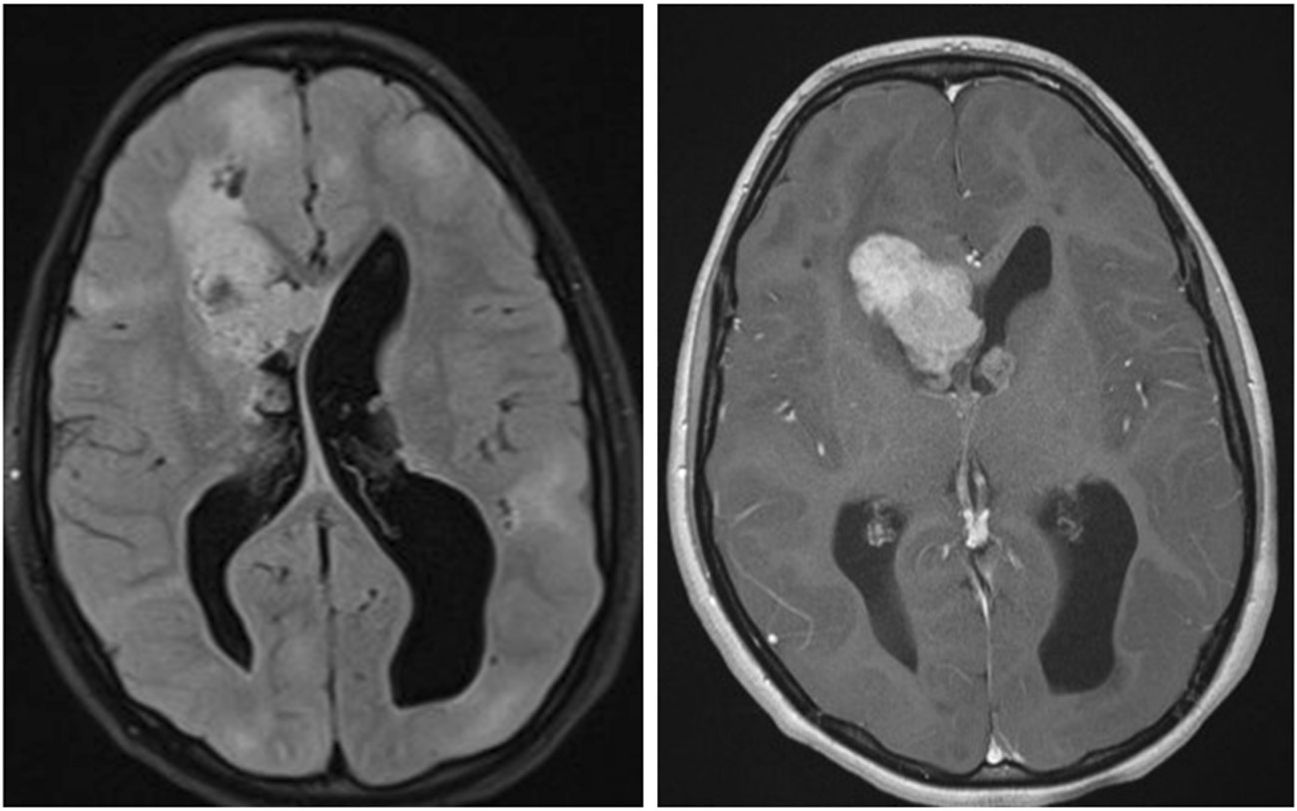
Subependymal giant cell astrocytoma (SEGA): axial FLAIR and T1-contrast-enhanced MRI of a 12-year-old girl with known tuberous sclerosis and epilepsy, presenting with new-onset symptoms of increased intracranial pressure. MRI shows multiple cortical tubers, and bilateral SEGA (prominent on the right side) with obstructive hydrocephalus. The child underwent transcortical resection of the tumor and a septostomy. No progression of the left tumor or recurrence of the right tumor is evident over a 6-year follow-up

Subependymal giant cell astrocytoma (SEGAs) are glioneuronal WHO grade I tumors. They occur in up to 15% of tuberous sclerosis (TS) patients [46]. Most SEGAs are located at the caudothalamic groove (CTG), abating the foramina of Monro (Fig. 5); however, lesions in other ventricular locations, and even outside the ventricular system, have been described as well [47, 48]. The origin of SEGA is assumed to be in the growth from subependymal nodules (SENs). However, in a large radiological series, it has been shown that SENs occur in various locations in the lateral ventricle, often with an attachment to the caudate nucleus, as opposed to SEGAs, which are located at the CTG [47]. On MRI, SEGAs are isointense on T1 and undergo a homogenous or heterogeneous enhancement. On CT, they often have a calcified component, usually at the attachment to the caudothalamic groove. SEN and SEGA share a similar histopathological appearance; however, differentiation between them is based on biological behavior and radiological appearance: SEGAs grow while SENs are stable, SEGAs are >1 cm in size, and SEGAs enhance following contrast injection [46].

The clinical impact of SEGA is usually associated with obstruction of the foramina of Monro, leading to obstructive hydrocephalus, with the signs and symptoms of increased intracranial pressure. As MRI scans are routinely performed for many TS patients (often due to seizures), many SEGAs are diagnosed incidentally, and not only while presenting with signs and symptoms of increased intracranial pressure [49]. In the past, surgery was the only effective treatment for SEGAs. However, over recent years, mammalian target of rapamycin inhibitors (mTORi) have been shown to be significantly effective in reducing SEGA size, as well as other TS-related manifestations, and are accepted as a disease-affecting drug [50].

Treatment options for SEGA include follow-up, resection, and mTORi [46, 50–52]. The debate over specific treatment indications, and the role of surgery as opposed to treatment with mTORi, is ongoing. According to the recommendations of the TS Consensus group from 2012, surgery is indicated for tumors causing acute deterioration from hydrocephalus or bleeding [46]. In other scenarios, mTORi are a valid alternative, especially if there are other TS findings such as multiple SEGAs, refractory seizures, or AML [53, 54]. However, evidence that mTOR inhibitors can be initiated in the context of hydrocephalus, even in the setting of acute symptoms of increased cranial pressure, has been shown as well [55]. Diagnosis of TS is based on various clinical criteria, as well as genetic alteration (of TS1 or TS2 genes) [49]. Diagnosis can be made based on a clinical/genetic diagnosis of TS and a typical tumor location without histology [46].

### Bifocal (suprasellar/pineal) germ cell tumor

Germ cell tumors (GCTs) account for less than 1% of all PBTs, occurring significantly more frequently in males, in Asians, and in the second decade of life [56–58]. GCTs are typically divided into germinomas and non-germinomas (embryonal carcinoma, yolk sac tumor, choriocarcinoma, and teratoma) with a predominance for germinomas [56]. Intracranial GCTs occur predominantly in the midline, around the third ventricle, with the suprasellar and pineal region being the most common locations [57]. Bifocal (suprasellar/pineal) germ cell tumors (BGCTs) are defined as a bifocal occurrence of GCTs in the suprasellar and pineal region. It is important to differentiate between “true” BGCTs (two separate focal lesions) and metastatic conditions (continuous spread from one region to the other) [57, 58]. The debate whether BGCTs are or are not always caused by metastatic spread is still ongoing; however, it seems that if no clear connection between the two lesions is seen on MRI, and in the absence of other lesions on the brain and spinal MRI, the lesions are to be considered a “true” BGCT [57].

**Fig. 6:**
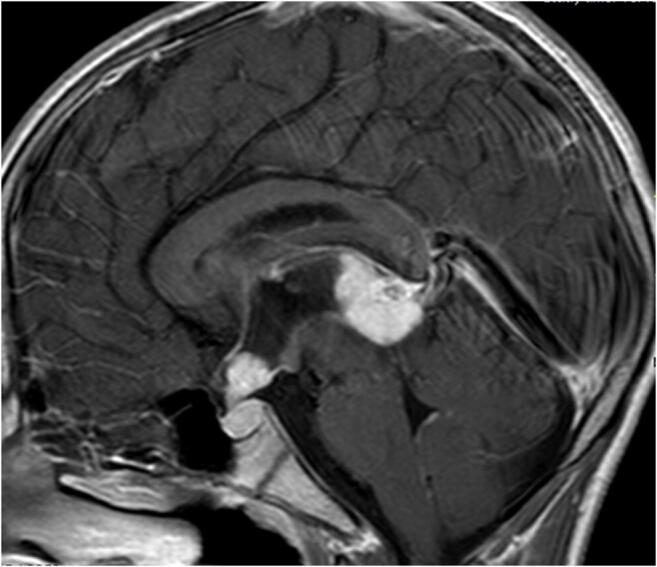
Bifocal (suprasellar/pineal) germ cell tumor (BGCT): sagittal T1-contrast-enhanced MRI of a 13-year-old boy presenting with obstructive hydrocephalus and diabetes insipidus. MRI shows a “bifocal–pineal and suprasellar” tumor. A shunt was placed. CSF and blood markers (AFP + BHCG) were negative. Patient was treated for a presumed germinoma and is tumor-free for more than 5 years. One year after shunt insertion, he underwent an endoscopic third ventriculostomy (ETV) due to a shunt malfunction

Some authors argue that biopsy of BGCT is not necessary, since its occurrence is practically pathognomonic of germinomas [57, 59]. However, others challenge this, since individual reports show that BGCTs are not restricted to germinomas [57, 58]. The distinction between germinomas and non-germinomas, or even other lesions such as pinealoblastoma [56], may be important, since the indications for surgery, radiation field, and chemotherapy regimens differ for the different tumor types. Some MRI features might help to distinguish between germinomas and non-germinomas. Pineal germinomas typically present as solid hypo- to isointense lesions on T1-weighted imaging and hyperintense lesions on T2-weighted imaging (Fig. 6). An infiltrative margin and the presence of a pure solid tumor are suggestive of a germinoma, and a homogenous contrast enhancement is often seen [58]. Most bifocal lesions are germinomas [58], and apparent diffusion coefficient (ADC) values are significantly lower in germinomas as opposed to non-germinomas [58]. Non-germinomas occur more often in the pineal region or cerebral hemispheres. They typically show heterogeneous contrast enhancement and intratumoral hyperintense foci on T1-weighted imaging [58]. Non-germinomas were shown to be significantly larger than germinomas [58]. Wu et al. showed that the combination of lack of hyperintense foci on T1-weighted imaging, mild or no contrast enhancement, and ADC under the threshold value (1.143 × 10^−3^ mm^2^/s) had a 100% sensitivity and 100% negative predictive value for discriminating germinoma from non-germinoma [58]. Gradient echo and SWI sequences are also very useful for differentiating germinoma and non-germinoma germ cell tumors [60]. Hemorrhages and cerebral hemiatrophy seem to be associated with ectopic germinomas and lead to unusual clinical and radiological presentations; however, they help narrowing down the diagnosis. Tumor markers such as beta human chorionic gonadotropin (BHCG) and alpha feto-protein can also help in differentiating between germinoma and non-germinoma, although these analyses are not specific, and only in 30–40% of the cases do germinomas secrete BHCG [61]. At times, germinomas arise from the cerebral parenchyma away from the midline structures, especially adjacent to the basal ganglia, thalamus, or internal capsule [62]. Based on the available data, it seems that when a lesion appears to be a BGCT, and the typical aspects of a germinoma are seen on MRI, diagnosis can be made with very high certainty and treatment can be initiated without prior biopsy. This is true even in cases where tumor markers are found to be normal. To state, that even for GCT in general, it is now well accepted in large cooperative groups (e.g., Society of Pediatric Oncology and Children’s Oncology Group) that in a patient with positive markers, upfront surgery is unnecessary. However, after initiating treatment, a follow-up MRI should be done in order to evaluate whether the tumor is responding to treatment. If not, the diagnosis needs to be revised and confirmed through endoscopic, stereotactic, or open biopsy.

## Trilateral retinoblastoma

Retinoblastoma (RB) is initiated by mutations in the *RB1* tumor suppressor gene [63]. Patients with *RB1* mutations also have about a 5% risk of developing intracranial midline primitive neuroectodermal tumors (e.g., pineoblastomas) [64]. The combination of bilateral RB and an embryonal tumor in a child is referred to as a trilateral retinoblastoma (TRB), occurring in 0.6–12.7% of patients with RB [63]. The tumors are usually found within the pineal region (75%) or sellar region (22%), rarely in the cerebellar region (1%) or in multiple regions (1%) [63]. The mean age at presentation is between 1 and 2 years of age [65]. Treatment with high-dose chemotherapy combined with stem-cell rescue helped improve survival rates, which are estimated at 44% after 5 years [64].

Diagnosis of RB is usually made by fundoscopy and ultrasound (US) [65]. In almost all cases, intratumoral calcifications are detected by US, increasing the confidence rate regarding diagnosis [65]. While examination of the children under general anesthesia using fundoscopy and US will almost inevitably lead to a diagnosis of RBF, MRI has proven to be the most sensitive technique [65] for evaluation of tumor infiltration of the optic nerve, extraocular extension, and detection of intracranial manifestations. Although imaging is used as the basis for diagnosis and treatment decisions of RB, the imaging modality and minimum quality of MRI are not standardized [65]. A detailed MRI protocol for the detection of brain lesion in RB patients is recommended by the European Retinoblastoma Imaging Collaboration (ERIC), while midline structures of the brain (pineal, sellar/parasellar, infratentorial regions) should be analyzed carefully [65]. MRI characteristics of TRB are heterogeneous and can present as completely solid, solid with a cystic component, or completely cystic with an irregularly thickened rim [66]. Cystic TRB might mimic normal pineal gland cysts, and vice versa. While TRBs show an irregular or thickened cyst wall, sometimes with tiny nodules, pineal cysts present with a thin wall (maximum 2 mm) with isointense central region of the cyst on T2-weighted images and isointense on T1-weighted images, as well as enhancement after contrast injection [66].

In the case of cystic TRBs, some authors recommend a three-group classification system:

1. Probably benign pineal cyst
2. Obvious cystic TRB
3. Suspicious pineal cyst

For the first group, a follow-up MRI after 6 months is recommended, while for the last group, closer follow-up (e.g., MRI after 3 months) is warranted.

TRBs are usually well defined, relatively isointense on T1- and T2-weighted images, contrast enhancement is mostly heterogeneous, and hydrocephalus is a typical complication of large lesions [66]. Physiological calcifications of the pineal gland under the age of 6 years are rare; therefore, any calcification of the pineal region at such an age is likely to be abnormal and should lead to further follow-up to exclude a pineal neoplasm [67]. Any pineal neoplasm presenting before the age of 4 years should lead to an ophthalmological examination to rule out a TRB [67]. In view of the rarity of RB, any pineal or sellar/parasellar neoplasm in a child with bilateral or familial RB is considered to be a TRB, without the need of further tissue diagnosis [67]. Although the diagnosis of TRB can be made based on imaging, at times (within the scope of research protocols, e.g., SJMB03), craniospinal radiation dose can be reduced following gross total or near total resection.

## Discussion

Diagnosing a brain tumor based solely on MRI scans has been the cornerstone of some intracranial lesions with pathognomonic radiological characteristics. However, over recent years, molecular profiling has entered into common practice, especially with respect to prognosis and treatment guidance, in what is often termed *personalized medicine*. Despite the increased role of molecular profiling, the debate on the need for tissue diagnosis in several tumors is still valid. In addition, for some pediatric tumors, such as embryonal tumors, in which imaging features are useful for the diagnosis, biopsy is still necessary and important for confirming the suspected diagnosis.

Radiomics, machine learning, and artificial intelligence (AI) have all been evaluated in the diagnosis of brain tumors, in both adults and children [68]. Through extensive computerized analysis of MRI scans, examples of differentiating between various histopathologies and even molecular profiles have been described [69–72]. Nevertheless, radiomics is still a research tool, and future implications for diagnosing and molecular profiling have yet to be determined. These technologies may eventually increase the spectrum of pathologies which may be diagnosed solely based on MRI, including molecular profiling of tumors, leading to neoadjuvant protocols without a prior biopsy [73].

Recently, studies of liquid biopsies obtained from blood, CSF, or urine for the detection of brain tumors have been presented [74–76]. Proteomics (e.g., circulating tumor cells), lipidomics (circulating tumor lipids), and metabolome (metabolized tumor products) are available for analysis and show promising results [76]. In the future, liquid biopsies might be a supplementary technique for typical MRI findings, or might even replace surgical biopsies for the diagnosis of brain tumors [74, 76]. However, to date, the statistical power of the available studies is not sufficient to provide firm recommendations for clinical use [74, 76].

Acoustic schwannomas in patients with NF2, radiation-induced meningiomas, dysembryoplastic neuroepithelial tumors (DNETs), and low-grade gliomas (LGGs)/incidentalomas are other entities that can be diagnosed based on typical radiological criteria. These lesions, however, are not discussed within the scope of this article since they either occur more often in adult patients (e.g., schwannoma in NF2 or radiation-induced meningioma) or since once they show some changes in radiological features, they will eventually need biopsy or resection to confirm the diagnosis and tailor further treatment (DNET/LGG/incidentalomas) [77, 78]. To note that many patients with NF2 are currently diagnosed earlier and treated during childhood, therefore, in the future, NF2-related tumors might be considered pediatric brain tumors, rather than tumors of adulthood [79, 80].

## Conclusion

The diagnosis of most PBTs must be confirmed through histopathology and molecular biology, achieved by surgical biopsy or resection. However, diagnosis based on typical MRI findings, in conjunction with the clinical presentation, is possible for a select group of PBTs. The role of a biopsy to rule out rare alternative diagnoses, as well as for medico-legal aspects, should be acknowledged. In selected cases, molecular profiling might significantly impact treatment and prognosis, justifying a biopsy within the scope of clinical trials, as the route to personalized medicine tailored to the specific tumor.
